# NVP-BEZ235 Attenuated Cell Proliferation and Migration in the Squamous Cell Carcinoma of Oral Cavities and p70S6K Inhibition Mimics its Effect

**DOI:** 10.3390/ijms19113546

**Published:** 2018-11-10

**Authors:** Cheng-Ming Hsu, Pai-Mei Lin, Hsin-Ching Lin, Yao-Te Tsai, Ming-Shao Tsai, Shau-Hsuan Li, Ching-Yuan Wu, Yao-Hsu Yang, Sheng-Fung Lin, Ming-Yu Yang

**Affiliations:** 1 Department of Otolaryngology, Chiayi Chang Gung Memorial Hospital and Chang Gung University College of Medicine, Chiayi 61363, Taiwan; scm0031@adm.cgmh.org.tw (C.-M.H.); yaote1215@gmail.com (Y.-T.T.); b87401061@gmail.com (M.-S.T.); 2 School of Traditional Chinese Medicine, College of Medicine, Chang Gung University, Taoyuan 33302, Taiwan; smbepigwu77@gmail.com (C.-Y.W.); r95841012@adm.cgmh.org.tw (Y.-H.Y.); 3 Department of Nursing, I-Shou University, Kaohsiung 82445, Taiwan; paimei@isu.edu.tw; 4 Department of Otolaryngology, Kaohsiung Chang Gung Memorial Hospital and Chang Gung University College of Medicine, Kaohsiung 83301, Taiwan; hclin@cgmh.org.tw; 5 Division of Hematology-Oncology, Department of Internal Medicine, Kaohsiung Chang Gung Memorial Hospital and Chang Gung University College of Medicine, Kaohsiung 83301, Taiwan; lee0624@cgmh.org.tw; 6 Department of Chinese Medicine, Chiayi Chang Gung Memorial Hospital, Chiayi 61363, Taiwan; 7 Division of Hematology and Oncology, Department of Internal Medicine, E-Da Hospital, Kaohsiung 82445, Taiwan; 8 School of Medicine, I-Shou University, Kaohsiung 82445, Taiwan; 9 Graduate Institute of Clinical Medical Sciences, College of Medicine, Chang Gung University, Tao-Yuan 33302, Taiwan

**Keywords:** oral cavity squamous cell carcinoma (OSCC), NVP-BEZ235, mTOR, p70S6K

## Abstract

NVP-BEZ235 or BEZ235 is a dual inhibitor of adenosine triphosphate (ATP)-competitive phosphoinositide 3-kinase (PI3K)/mammalian-target-of-rapamycin (mTOR) and is promising for cancer treatment. Because it targets more than one downstream effector, a dual approach is promising for cancer treatment. The aim of this study was to evaluate the efficacy of NVP-BEZ235 in treating oral cavity squamous cell carcinoma (OSCC). Two human OSCC cell lines, SCC-4 and SCC-25, were used in this study. PI3K-AKT signaling, proliferation, and cell migratory and invasion capabilities of OSCC cells were examined. In NVP-BEZ235-treated SCC-4 and SCC-25 cells, the phosphorylation of 70-kDa ribosomal S6 kinase (p70S6K), but not mTOR, decreased within 24 h. NVP-BEZ235 inhibited OSCC-cell proliferation, migration, and invasion possibly by directly deregulating the phosphorylation of p70S6K. The phospho-p70S6K inhibitor mimicked the effects of NVP-BEZ235 for preventing proliferation and weakening the migratory and invasion abilities of SCC-4 and SCC-25 cells. This study further confirmed the effect of NVP-BEZ235 on OSCC cells and provided a new strategy for controlling the proliferation, migration, and invasion of OSCC cells using the phopho-p70S6K inhibitor.

## 1. Introduction

Squamous cell carcinoma of the head and neck (SCCHN) is the sixth most common malignancy worldwide. With a mortality rate of approximately 50%, it affects 600,000 new patients every year. Oral cavity squamous cell carcinoma (OSCC) accounts for the vast majority of all SCCHN cases [1]. OSCC has the sixth highest cancer incidence in Taiwan and is the most common malignancy diagnosis for Taiwanese men aged 30 to 50 years [2,3]. Most treatment modalities are based on tumor (T) staging, and they include surgery and adjuvant therapy, such as chemotherapy and radiotherapy [4].

Even though progress has been made in cancer treatment, oral cancer has high rates of local recurrence, secondary primary malignancy, and morbidity [5]. Once patients with inoperable and recurrent OSCC, or distant metastasis, platinum-combination therapy is the standard first-line treatment [6,7]. However, if cis-diamminedichloridoplatinum (CDDP)-based chemotherapy fails and a patient’s cancer is still inoperable, the therapeutic options are limited; moreover, most patients are only eligible to receive palliative radiation or supportive care [8,9,10].

With the advances of cancer research, target therapy has become the major trend for various malignant diseases as the first- or second-line treatment option, including OSCC [11]. Synergistic antitumor effects exerted by combination of targeted therapy with CDDP have been demonstrated in many preclinical studies [12,13]. The PI3K/AKT/mTOR intracellular signaling pathway plays a vital role in various physiological processes, such as cellular survival, migration, proliferation, and differentiation, as well as angiogenesis, protein synthesis, and glucose metabolism. Additionally, the PI3K/AKT/mTOR pathway is associated with various oncogenic processes, and is one of the signaling pathways most frequently dysregulated in cancer, including OSCC [14]. The PI3K/AKT/mTOR pathway and its downstream 70-kDa ribosomal S6 kinase (p70S6K) are constitutively activated in human tumor cells, providing unique opportunities for therapeutic intervention. Therefore, targeting PI3K/AKT/mTOR signaling could be a rational strategy for the treatment of OSCC, a disease—particularly when advanced—in which systemic therapy plays a crucial role. The ability of NVP-BEZ235 (dactolisib), a dual PI3K/mTOR inhibitor, to treat some cancer types is being evaluated in phase I/II clinical trials. NVP-BEZ235 is an imidazo [4] quinoline derivative. It binds to the ATP-binding cleft of enzymes and thus inhibits PI3K and mTOR kinase activity [5]. A dual approach that targets more than one downstream effector is promising because it may delay or even prevent therapy resistance [15]. NVP-BEZ235 has exhibited antitumor effects on lung cancer [16,17], human glioma cells [18,19], breast cancer [20,21], melanoma [22], pancreatic cancer [23,24], sarcoma [15,25], nasopharyngeal cancer [26,27], and hepatoma [28,29,30]. Additionally, NVP-BEZ235 demonstrated great promise for controlling solid tumors in a preclinical mouse model [15].

The p70S6K is a member of the protein kinase A, G, and C families (AGC) serine/threonine kinase family which contains more than 60 human proteins including Akt, protein kinase C, and 90-kDa ribosomal S6 kinase [31]. By increasing ribosomal production and mRNA translation, p70S6K can promote cell growth through global protein synthesis [31]. p70S6K is a downstream target of the mTOR signaling pathway, specifically mTOR complex 1 (mTORC1). p70S6K is also a downstream signal of mitogen activated protein kinase (MAPK)/extracellular-signal-regulated kinase (ERK) pathway. p70S6K involves in the cross-talk between mTOR and MAPK/ERK signaling pathways at various regulatory levels. Activation of p70S6K occurs via phosphorylation at serine-411 (Ser411), threonine-421 (Thr421), and Ser424 by endogenous mitogens such as epidermal growth factor, thrombin, and lysophosphatidic acid. The p70S6K pathway is also essential for signaling two filamentous actin (F-actin) microdomains in cells and regulating cell migration [32].

In the present study, we investigated the effect of NVP-BEZ235 on PI3K/AKT/mTOR signaling in OSCC cells in vitro. We first discovered that NVP-BEZ235 inhibited proliferation and attenuated cell migration in a subset of SCC-4 and SCC-25 cells and thus enhanced reduction of p70S6K expression. We also used a p70S6K inhibitor to investigate the possibility of substituting NVP-BEZ235, which has undesirable side effects.

## 2. Results

### 2.1. Analysis of mTOR Expression in OSCC Tissue Using Real-Time Quantitative Reverse Transcriptase—Polymerase Chain Reaction (qRT-PCR)

**Figure 1:**
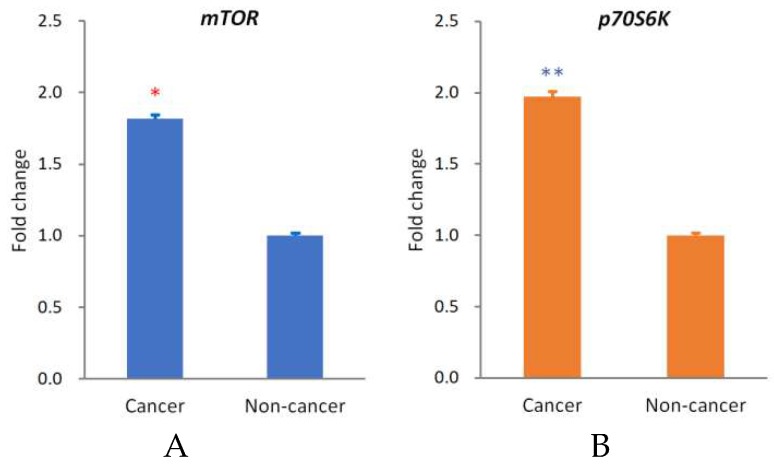
Expression *of mTOR* and *p70S6K* of squamous cell carcinoma (OSCC). Expression of *mTOR* (**A**) and *p70S6K* (**B**) was upregulated in the cancerous tissue of OSCC (*p* < 0.05 and 0.01, respectively). The *y*-axis represents the fold change in the *mTOR* or *p70S6K* expression level of cancerous relative to noncancerous tissues. The mean *mTOR* or *p70S6K* expression level in noncancerous tissues was assigned a value of 1 to obtain the fold change in expression in cancerous tissues. The mean ΔCt values for *mTOR* are 5.98 ± 0.26 (cancer parts) and 6.84 ± 0.22 (noncancer parts), and for *p70S6K* are 6.03 ± 0.31 (cancer parts) and 7.01 ± 0.19 (noncancer parts).The red * *p* < 0.05 and blue ** *p* < 0.01 indicate the statistical significance of differences between the cancer parts and noncancer parts.

To clarify whether the expression levels of *mTOR* and *p70S6K* were different in cancerous tissue compared with noncancerous tissue, cancerous and noncancerous tissue samples taken from the 28 OSCC patients were examined using qRT-PCR to determine the expression of *mTOR* and *p70S6K*. Our data demonstrated that the expression levels of mTOR (*p* < 0.05) and p70S6K (*p* < 0.01) were significantly upregulated in OSCC (Figure 1A).

### 2.2. NVP-BEZ235 Inhibited Cell Proliferation and Downregulated the PI3K/AKT/mTOR-Signaling Pathway of OSCC Cells, Resulting in the Suppression of Phospho-p70S6K

**Figure 2:**
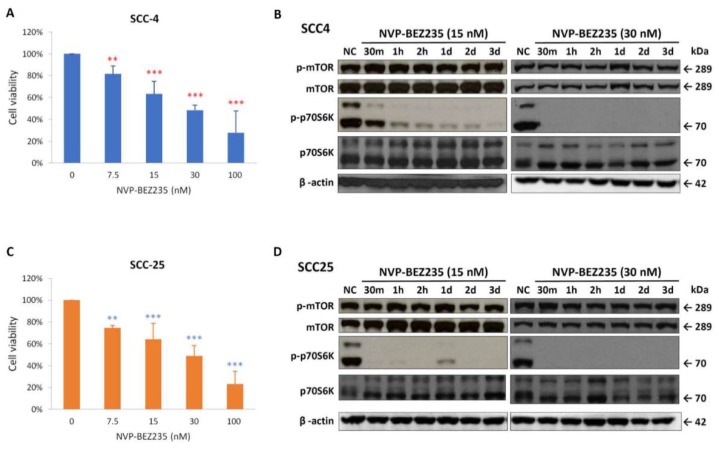
NVP-BEZ235 suppressed cell proliferation and reduced the expression of phospho-mTOR and phospho-p70S6K in SCC-4 and SCC-25 cells. The inhibitory effects of various doses (7.5, 15, 30, and 100 nM) of NVP-BEZ235 on SCC-4 (**A**) and SCC-25 (**C**) cells were assessed using MTT assay after 72 h of treatment. Data presented are the mean and standard error of the mean of three independent experiments. The ** *p* < 0.01 and *** *p* < 0.001 indicate the statistical significance of differences between the results for cells with and without treatment (red ** and *** for SCC-4 and blue ** and *** for SCC-25). As determined through Western blotting, NVP-BEZ235 reduced the expression of phospho-mTOR (p-mTOR) and phospho-p70S6K (p-p70S6K) in SCC-4 (**B**) and SCC-25 (**D**) cells. SCC-4 and SCC-25 cells were treated with 15 or 30 nM NVP-BEZ235 for 30 min (30 min), 1 h (1 h), 2 h (2 h), 1 day (1 d), 2 days (2 d), and 3 days (3 d) in six-well plates. Western blot analysis was performed to examine the expression levels of p-mTOR, mTOR, p-p70S6K, p70S6K, and β-actin.

The antiproliferative potential of NVP-BEZ235 was assessed using 3-(4.5-dimethylthiazol-2-yl)-2,5-diphenyl-tetrazolium bromide (MTT) assay on SCC-4 and SCC-25 cells. After 72 h of treatment, NVP-BEZ235 had significantly inhibited the growth of SCC-4 (Figure 2A) and SCC-25 (Figure 2C) when it was used at concentrations of 7.5 nM and greater. The phosphorylation of p70S6K decreased within 24 h, and the phosphor-p70S6K was completely absent for at least 3 days when the 30 nM dose was administered (Figure 2B,D). However, the phosphorylation of mTOR did not reduce significantly up to 3 days.

### 2.3. NVP-BEZ235 Inhibited the Migratory and Invasion Abilities of SCC-4 and SCC-25 Cells

**Figure 3:**
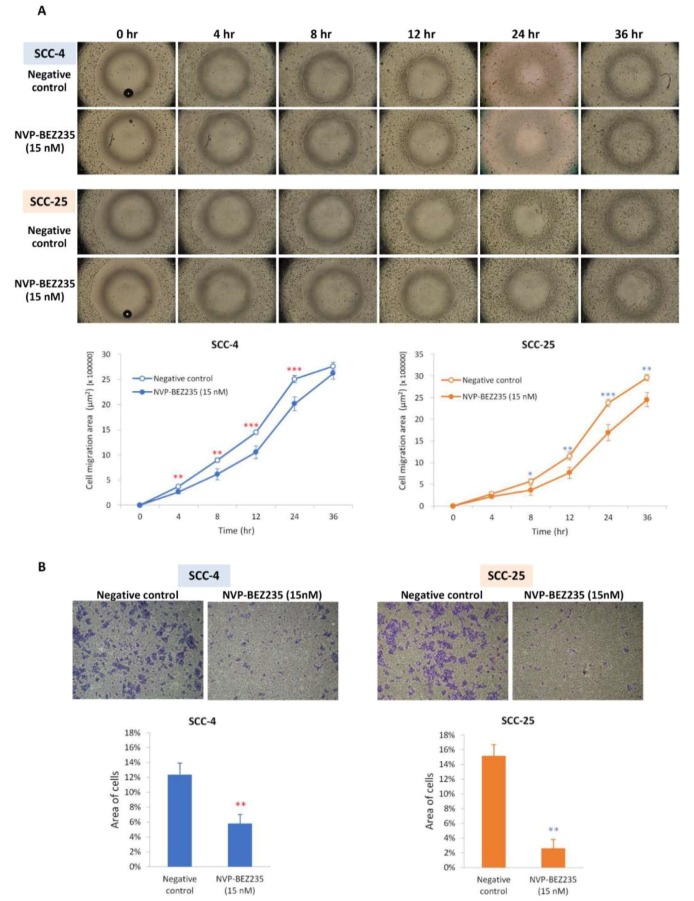
NVP-BEZ235 inhibited the migratory and invasion capabilities of SCC-4 and SCC-25 cells. (**A**) Wound-healing assay determined that SCC-4 and SCC-25 cells had shorter migration distances after NVP-BEZ235 treatment. * *p* < 0.05, ** *p* < 0.01, and *** *p* < 0.001 indicate the statistical significance of differences at one point in time between the results for cells with and without treatment. Hollow dots are for negative controls and solid dots are for NVP-BEZ235 treatments. (**B**) After incubating for 24 h (SCC-4) or 48 h (SCC-25) with transwell chambers, the area of migratory or invasive cells was markedly decreased in NVP-BEZ235-treated cells in comparison with cells without treatment. ** *p* < 0.01 indicates the statistical significance of the differences between cells with and without treatment (red ** for SCC-4 and blue ** for SCC-25).

Weaker migratory ability was observed in SCC-4 and SCC-25 cells that had been treated with NVP-BEZ235 through the detection of the wound-healing assay (Figure 3A). In SCC-4 cells, migration was significantly slower in the cells that had been treated with NVP-BEZ235 for 4 to 24 h than in untreated cells. In SCC-25 cells, migration was significantly slower from 8 to 36 h after NVP-BEZ235 treatment. Weaker invasion ability was also observed in SCC-4 and SCC-25 cells that had been treated with NVP-BEZ235, as detected using the transwell cell migration assay (Figure 3B). After incubation for 24 h in transwell chambers, the number of cells that had migrated or invaded was markedly decreased in NVP-BEZ235-treated SCC-4 (*p* < 0.01) and SCC-25 (*p* < 0.01) cells.

### 2.4. Phospho-p70S6K Inhibitor 2-((4-(5-Ethylpyrimidin-4-yl)piperazin-1-yl)methyl)-5-(trifluoromethyl)-1H-benzo[d]imidazole (PF-4708671) Suppressed Proliferation and Inhibited the Expression of Phospho-mTOR and Phospho-p70S6K in SCC-4 and SCC-25 Cells

**Figure 4:**
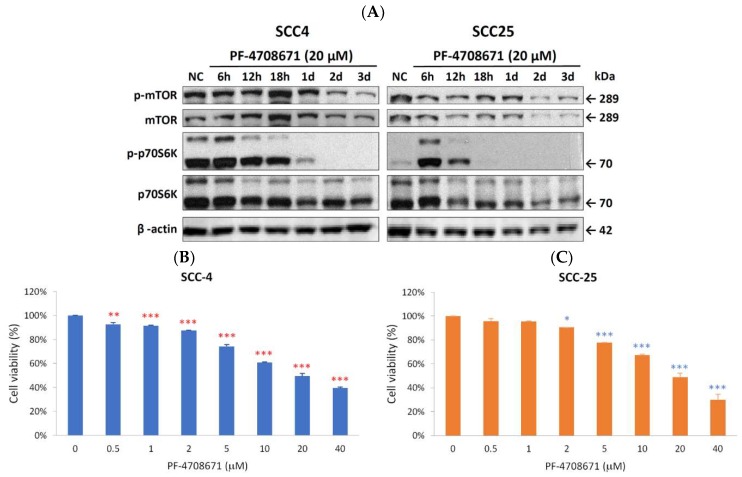
Phospho-p70S6K inhibitor (PF-4708671) suppressed phospho-p70S6K expression and cell proliferation in SCC-4 and SCC-25 cells. (**A**) As determined using Western blotting, the phospho-p70S6K (p-p70S6K) inhibitor (PF-4708671) reduced the expression of phospho-mTOR (p-mTOR) and p-p70S6K in SCC-4 and SCC-25 cells. SCC-4 and SCC-25 cells were not treated as negative control (NC) or treated with 20 µM PF-4708671 for 6 h (6 h), 12 h (12 h), 18 h (18 h), 1 day (1 d), 2 days (2 d), and 3 days (3 d). Western blot analysis was performed to examine the expression levels of p-mTOR, mTOR, p-p70S6K, p70S6K, and β-actin. (**B**) Inhibitory effects of various doses (0.5, 1, 2, 5, 10, 20, and 40 µM) of PF-4708671 on SCC-4 (**B**) and SCC-25 (**C**) cells were assessed using MTT assay after 72 h of treatment. Data presented are the mean and standard error of the mean of three independent experiments. * *p* < 0.05, ** *p* < 0.01, and *** *p* < 0.001 indicate the statistical significance of differences between cells with and without treatment. t (red ** and *** for SCC-4 and blue *, ** and *** for SCC-25).

Because NVP-BEZ235 did not demonstrate proliferative abilities through phospho-p70S6K inhibition, we wondered if the direct suppression of phospho-p70S6K would achieve the same effect. PF-4708671, a phospho-p70S6K inhibitor, was used to evaluate its antiproliferative potential in SCC-4 and SCC-25 cells. As displayed in Figure 4A, the expression of phospho-p70S6K was completely abolished in SCC-4 (2 days) and SCC-25 (18 h) after PF-4708671 treatment. The expression of phosphor-mTOR was also downregulated by PF-4708671 treatment. MTT assays were also performed to determine the effects of PF-4708671 on cell growth. After 72 h of PF-4708671 treatment, the growth of SCC-4 and SCC-25 cells was significantly inhibited (Figure 4B). SCC-4 cells were more sensitive to PF-4708671 than SCC-25 cells were.

### 2.5. Phospho-p70S6K Inhibitor (PF-4708671) also Suppressed Migration and Invasion as an NVP-BEZ235 in SCC-4 and SCC-25 Cells

**Figure 5:**
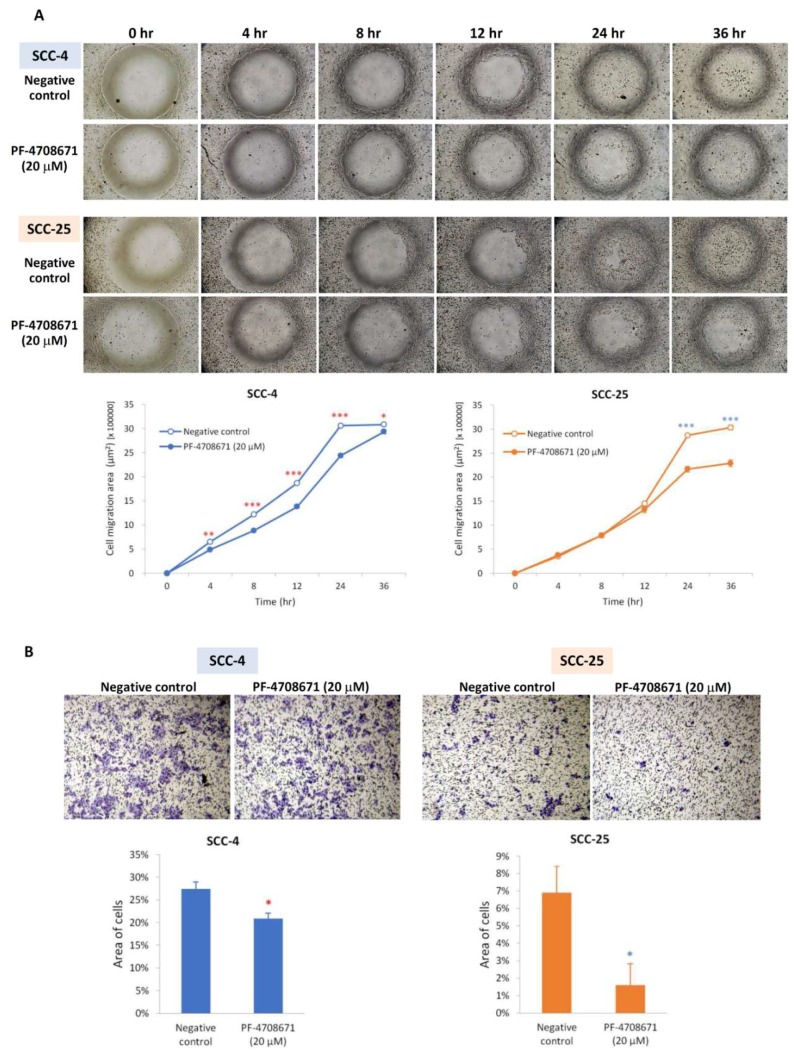
Phospho-p70S6K inhibitor (PF-4708671) inhibited the migratory and invasion activities of SCC-4 and SCC-25 cells. (**A**) Wound-healing assay revealed that SCC-4 and SCC-25 cells had shorter migration distances after PF-4708671 treatment. * *p* < 0.05, ** *p* < 0.01, and *** *p* < 0.001 indicate the statistical significance of the differences at one point in time between cells with and without treatment. (**B**) After incubating for 24 h (SCC-4) or 48 h (SCC-25) in transwell chambers, the area of migratory or invasive cells was markedly decreased in PF-4708671-treated cells compared with cells without treatment. * *p* < 0.05 indicates the statistical significance of differences between cells with and without treatment (red * for SCC-4 and blue * for SCC-25).

The migratory and invasion abilities of SCC-4 and SCC-25 cells were also weakened by the phospho-p70S6K inhibitor (PF-4708671). In SCC-4 cells, migration was significantly slower in cells treated with PF-4708671 for 4 to 36 h than in untreated cells. In SCC-25 cells, migration was significantly slower at 24 and 36 h after PF-4708671 treatment (Figure 5A). After incubating for 24 h in transwell chambers, the number of migrated and invaded cells was markedly decreased in PF-4708671-treated SCC-4 (*p* < 0.05) and SCC-25 (*p* < 0.05) cells (Figure 5B).

## 3. Discussion

This is the first study to investigate the effect of NVP-BEZ235 therapy in OSCC. NVP-BEZ235 is a novel, orally consumable dual PI3K/mTOR inhibitor that is currently being used in clinical trials [33]. The PI3K/AKT/mTOR signaling pathway and abnormal activation of this pathway reportedly play an essential role in the progression, metastasis, and chemoresistance of numerous tumor types [34]. Currently, NVP-BEZ235 is in phase I/II clinical trials and was demonstrated to control solid tumors in a preclinical mouse model [33].

In our patients with OSCC, the expression of *mTOR* and *p70S6K* was significantly upregulated (Figure 1). It has been reported that p70S6K plays an important role in metastasis within the mTOR signaling networks, including mTORC1 and mTORC2 [35]. Our in vitro results demonstrated that NVP-BEZ235 significantly reduced SCC-4 and SCC-25 proliferation. They also revealed that NVP-BEZ235 suppressed phospho-mTOR and phospho-p70S6K levels. S6 kinase proteins (S6K) has also been reported to influence apoptosis through different mechanisms [36,37]. In the PI3K/AKT/mTOR pathway, activation of mTOR results in the phosphorylation of numerous substrates, including the phosphorylation of S6K by mTORC1. The effect of NVP-BEZ235 on the apoptosis of OSCC cells may be associated with the phosphorylation of S6K. The antitumor effects of NVP-BEZ235 result not only from inhibiting the Akt survival pathway but also from promoting cell apoptosis. These effects raise the possibility that a combination treatment, once developed, would be a promising therapeutic strategy for enhancing the effects of chemotherapy and improving clinical outcomes for patients with OSCC. NVP-BEZ235 completely reduces phosphor-p70S6K activation and can inhibit phospho-mTOR activation. p70S6K has been reported to regulate cytoskeletal organization and cell motility induced by members of the Ras homologous (Rho) GTPase family, such as Ras homolog gene family, member A (Rho A), Ras-related C3 botulinum toxin substrate 1 (Rac1), and cell division control protein 42 homolog (cdc42) [38]. Therefore, NVP-BEZ235 affects not only cell proliferation but also cell migration (Figure 3). The rate of distant metastasis or regional lymph node metastasis of OSCC is possibly reduced by NVP-BEZ235.

The function of PI3K/Akt pathway is to promote cell survival and to inhibit apoptosis. When the intracellular signaling of PI3K/Akt pathway is altered, the cellular proliferation will be promoted and the upregulated glycolysis caused by the Warburg effect will be used to sustain the higher metabolic demand of transformed cells [39]. Carlo et al. reported grade 3–4 adverse effects of NVP-BEZ235 in 50% of patients (5 of 10) [40], without objective responses from subjects in the study group. The fatigue, diarrhea, nausea, and mucositis that has been reported with NVP-BEZ235 has limited the doses in which it is commonly prescribed, and it is unsurprising that combined PI3K and mTOR blockades resulted in frequent adverse effects [41]. Hence, caution is advised when taking NVP-BEZ235 orally.

According to our results, the p70S6K inhibitor could mimic the effects of NVP-BEZ235 and other mTOR inhibitors. The phospho-p70S6K inhibitor significantly inhibited the growth of SCC-4 and SCC-25 cells (Figure 4). Therefore, the phosphor-p70S6K inhibitor could weaken the Warburg effect and replace the mTOR inhibitor in the future. Indeed, the phospho-p70S6K inhibitor (PF-4708671) could suppress not only phospho-p70S6K but also phospho-mTOR, which is a result superior even to that obtained with NVP-BEZ235 (Figure 4).

In conclusion, proliferation and migration of OSCC cells could be effectively inhibited by NVP-BEZ235 through direct deregulation of phosphorylation of p70S6K. Even p70S6K is the downstream of PI3K/AKT/mTOR pathway, inhibition of phospho-p70S6K could still reduce the phosphorylation of mTOR. This study further confirmed the effect of NVP-BEZ235 on OSCC cells and provided a new strategy for controlling the migration and proliferation of OSCC cells by using the phopho-p70S6K inhibitor.

## 4. Materials and Methods

### 4.1. Patients and Samples

This study enrolled 28 patients (27 men and one female aged 31–75 years; mean ± standard deviation [SD]: 53.23 ± 10.98 years) diagnosed with OSCC who underwent surgery in the Department of Otolaryngology at Kaohsiung Chang Gung Memorial Hospital between 2009 and 2012. The clinical pathological characteristics—such as age; sex; tumor, neck lymph node, and metastasis staging; tumor size; and survival—of the patients are listed in Table 1. Tissues of tumor and adjacent nontumor parts were obtained from surgery and tissue samples were snap-frozen in liquid nitrogen immediately after resection. Prior to tissue acquisition, all the patients agreed and signed the informed consent. The Institutional Review Board of the Kaohsiung Chang Gung Memorial Hospital approved this study on August 01, 2012 (IRB No. 100-4455A3).

### 4.2. Real-time Quantitative Reverse-transcriptase Polymerase Chain Reaction (qRT-PCR) Analysis

The total RNA of SCC-4 cells, SCC-25 cells, and cancerous and noncancerous tissues obtained from the patients with OSCC was extracted using a TRIzol reagent (Invitrogen; Life Technologies, Carlsbad, CA, USA), and the High-Capacity cDNA Reverse Transcription Kit (Applied Biosystems, Foster City, CA, USA) was used to synthesize complementary DNA (cDNA). The PCR reaction mixture contained 25 ng of cDNA; 0.5 μL of *mTOR* gene-expression assay (Hs00234508_m1, Applied Biosystems, Foster City, CA, USA) or β-actin (*ACTB*) gene-expression assay (Hs01060665_g1, Applied Biosystems, Foster City, CA, USA); and 5 μL of 2× TaqMan Universal PCR Master Mix (Applied Biosystems, Foster City, CA, USA). qPCR analysis was run in an ABI 7500 Fast Real-Time System (Applied Biosystems, Foster City, CA, USA), and the thermal parameters were 1 cycle of 95 °C for 10 min and 40 cycles of 95 °C for 20 s and 60 °C for 1 min. The threshold cycle (Ct) of the *mTOR* gene or *p70S6K* gene was first normalized to the *ACTB* internal control to obtain the relative threshold cycle (Δ*C*t), and then the 2^−ΔΔ*C*t^ method was used to calculate the relative expression of target gene.

### 4.3. Cell Culture

The two human SCCHN cell lines, SCC-4 and SCC-25, used in this study were purchased from the Food Industry Research and Development Institute in Taiwan. Both SCC-4 and SCC-25 cells are tongue squamous cell carcinoma. Cells were preserved and grown in a minimum essential medium (MEM)-F12 medium (Invitrogen, Life Technologies, Carlsbad, CA, USA) containing 0.4 μg/mL hydrocortisone (Sigma Aldrich, St. Louis, MO, USA) and 10% fetal bovine serum at 37 °C with 5% CO_2_.

### 4.4. MTT Assay

The mitochondrial conversion of MTT to formazine was used to determine the percentage of metabolically active cells. Various concentrations of NVP-BEZ235 were used to treat SCC-4 and SCC-25 cells. Cells treated with phosphate-buffered saline (PBS) were used as negative control. The culture media were replaced with Dulbecco’s Modified Eagle Medium/Nutrient Mixture F-12 (DMEM/F-12) (without phenol) containing 0.02% MTT (Sigma Aldrich, St. Louis, MO, USA) after different incubation times. After incubation for 4 h, the media containing MTT were then replaced with dimethyl sulfoxide (200 μL per well). The absorbance at a wavelength of 595 nm were measured using a DTX880 Multimode Detector (Beckman Coulter, Brea, CA, USA).

### 4.5. Western Blotting

Radioimmunoprecipitation assay buffer (20 mM Tris-HCl at pH 7.5, 150 mM NaCl, 1 mM Na2EDTA, 1 mM ethylene glycol tetraacetic acid (EGTA), 1% Nonidet P-40 (NP-40), 1% sodium deoxycholate, 2.5 mM sodium pyrophosphate, 1 mM β-glycerophosphate, 1 mM Na3VO4, and 1 μg/mL leupeptin) was added to samples for protein extraction. For Western blotting, 30 µg of the total lysates was separated using 6% to 15% sodium dodecyl sulfate–polyacrylamide gel electrophoresis and transferred to a polyvinylidene fluoride membrane (Millipore, Darmstadt, Germany). After blocking with dried nonfat milk for 1 h, the membrane was incubated overnight with primary antibodies at 1:3000 dilution. The primary antibodies and antibodies against phosphorylated epitopes used in this study were mTOR, phospho-mTOR (Ser2448), p70S6K, and phospho-p70S6K (all purchased from Cell Signaling Technologies, Danvers, MA, USA). β-actin (1:5000 dilution; Sigma Aldrich, St. Louis, MO, USA) was used as the internal control. Horseradish-peroxidase-conjugated goat anti-mouse IgG (Sigma Aldrich, St. Louis, MO, USA) and goat anti-rabbit Immunoglobulin G (IgG) (Sigma Aldrich, St. Louis, MO, USA) were used as secondary antibodies. Western Lightning^®^ Plus-Enhanced Chemiluminescence (ECL) Substrates (PerkinElmer, Inc., Boston, MA, USA) were used to visualize the proteins.

### 4.6. Wound-Healing Assay

The migration activity of cells was analyzed using wound-healing assay. Cultures of SCC-4 and SCC-25 cells were optimized to ensure a homogeneous and viable cell monolayer prior to application of the wound-healing assay. One day before the assay, 2 × 10^5^ cells were seeded in 6-well plates, and when cell confluence reached approximately 90%, a homogeneous wound was artificially created on the monolayer using a sterile, plastic, 200-µL-micropipette tip. After creating the wound, cells were washed with PBS to remove debris. Cells that had migrated into the wounded area were photographed using a Zeiss microscope (Zeiss, Gottingen, Germany) at 40× magnification, and the migration area was calculated using ImageJ free software, version 1.41o (NIH, Bethesda, MD, USA).

### 4.7. Transwell Assays

The migration ability of SCC-4 and SCC-25 cells was measured using a 24-pore transwell chamber (Corning Inc., Corning, NY, USA) with a polycarbonate membrane filter covered by a gelatin package. The bottom membranes (8-μm aperture) of the transwell chambers were coated with Matrigel (Sigma, St. Louis, MO, USA) for the determination of invasive ability. Cells (5 × 10^5^ in 200 μL) were inoculated onto the upper chamber, and the lower chamber was filled with 600 μL of DMEM nutrient solution containing 10% fetal bovine serum (FBS). After a 24- to 48-h incubation at 37 °C with 5% CO_2_, the wells were removed, fixed with methanol and glacial acetic acid (3:1), stained with 0.1% crystal violet, and finally mounted [33]. The areas of migratory or invasive cells were discerned by calculating five randomly selected fields of stained cells using ImageJ free software, version 1.41o (NIH, Bethesda, MD, USA) [42].

### 4.8. Statistical Analysis

The data sets for MTT assay, wound-healing assay, and transwell migration assay consisted of at least three biological replicates, and the data are expressed as a mean ± SD. For statistical analysis of the gene expression of qRT-PCR, ΔCt values were used. The statistical significance was determined using a two-sample *t*-test, and *p*-values < 0.05 mean if null hypotheses of no difference were rejected. All the statistical analyses in this study were performed using SPSS version 15.0 software (SPSS, Chicago, IL, USA).

## Figures and Tables

**Table 1:** Characteristics of patients with OSCC.

Characteristic	Number of Patients
Sex	
Male	27
Female	1
Median age year (range)	53.23 (31–75)
Staging ^1^	
I	6
II	6
III	7
IV	9
Site	
Bucca	7
Gum	6
Palate	1
Tongue	12
Trigone	2
N stage ^1^	
N0	20
N1	7
N2a	0
N2b	1
N2c	0
N3	0
Survival	
Expired	10 ^2^
Survived	18

^1^ The stage of OSCC is defined by the National Comprehensive Cancer Network (NCCN) clinical practice guideline 7th edition; ^2^ The patients died from disease after 5 years of follow-up.

## Abbreviations

CDDP	Cis-diamminedichloridoplatinum
cDNA	Complementary DNA
mTOR	Mammalian target of rapamycin
mTORC1	Mammalian target of rapamycin complex 1
MTT	3-(4,5-dimethylthiazol-2-yl)-2,5-diphenyl-tetrazolium bromide
OSCC	Oral cavity squamous cell carcinoma
p70S6K	70-kDa ribosomal S6 kinase
PBS	Phosphate-buffered saline
PI3K	Phosphoinositide 3-kinase
qRT-PCR	Real-time quantitative reverse transcriptase–polymerase chain reaction
S6K	S6 kinase proteins
SCCHN	Squamous cell carcinoma of the head and neck
